# Tribo-oxide Competition and Oxide Layer Formation of Ti_3_SiC_2_/CaF_2_ Self-Lubricating Composites during the Friction Process in a Wide Temperature Range

**DOI:** 10.3390/ma14237466

**Published:** 2021-12-06

**Authors:** Rui Zhang, Wei Feng, Fuyan Liu

**Affiliations:** 1 School of Mechanical Engineering, Chengdu University, Chengdu 610106, China; fengwei@cdu.edu.cn; 2 Sichuan Province Engineering Technology Research Center of Powder Metallurgy, Chengdu University, Chengdu 610106, China; 3 Institute for Advanced Materials Deformation and Damage from Multi-Scale, Chengdu University, Chengdu 610106, China; 4 School of Mechanical Engineering, Xinjiang University, Urumqi 830000, China; 5 School of Chemical Engineering and Materials, Changzhou Institute of Technology, Changzhou 213032, China; liufy@czu.cn

**Keywords:** Ti_3_SiC_2_, mechanical properties, friction and wear properties, tribo-oxidation, wear mechanism, oxide competition

## Abstract

Ti_3_SiC_2_/CaF_2_ composites were prepared by the spark plasma sintering (SPS) process. Both the microstructure of Ti_3_SiC_2_/CaF_2_ and the influence of test temperature on the tribological behavior of the Ti_3_SiC_2_/CaF_2_ composites were investigated. The synergistic effect of friction and oxidation was evaluated by analyzing the worn surface morphology. The results showed that Ti_3_SiC_2_/CaF_2_ were still brittle materials after adding CaF_2_, which was in agreement with Ti_3_SiC_2_. The hardness, relative density, flexural strength and compressive strength of the Ti_3_SiC_2_/CaF_2_ composites were slightly lower than those of Ti_3_SiC_2_, and the addition of CaF_2_ decreased the decomposition temperature of Ti_3_SiC_2_ from 1350 to 1300 °C. Simultaneously, as the temperature of the test increased, the friction coefficient of Ti_3_SiC_2_/CaF_2_ showed a downward trend (from 0.81 to 0.34), and its the wear rate was insensitive.

## 1. Introduction

MAX phase ceramics have received widespread attention owing to their having properties of both ceramics and metals [1,2]. The term “M_n+1_AX_n_ phase” was first used by Barsoum [2]. The general formula M_n+1_AX_n_ was abbreviated to MAX. The Ti_3_SiC_2_ ceramic was one of the representative MAX ceramic materials [3,4,5,6,7]. Although it showed the duality of tribology, its self-lubricating properties were not obvious [8,9,10]. Related research found that doping the hard phase or lubricating phase to improve the tribological properties of Ti_3_SiC_2_ was effective [11,12,13,14,15,16,17]. However, like solid lubricants of graphite or MoS_2_, it can be severely oxidized in a high-temperature atmosphere, restricting its use in lubrication above 500 °C [18].

Fluoride had high melting point, good chemical stability and thermal stability. It is still very stable in both high-temperature, extremely oxidative atmospheres, and high-temperature, extremely reducing atmospheres [19,20,21], and it is not easy to oxidize and decompose. Moreover, it softens and forms ductile phases at high temperatures [22]. In the research of high-temperature-tolerant self-lubricating composite materials, fluoride has always been the first choice for solid lubricants. After fluorides undergo the transition from toughness to brittleness, they can form a stable separating film with low shear on the friction surface, allowing them to effectively exert their lubricating properties in the working environment [23,24,25]. Among them, CaF_2_ is a high-temperature solid lubricant with excellent performances. It had good chemical stability at 25–1000 °C. Additionally, it shows good high temperature lubrication performance due to the change from brittleness to plasticity during mechanical friction at above 500 °C. It is thought that the good friction and wear performance of this material is mainly owed to CaF_2_ forming a solid lubricating film on the surface in a high temperature environment.

In this paper, by using CaF_2_ as a solid lubricant, a Ti_3_SiC_2_-based self-lubricating composite (abbreviated as TSC-CF) was synthesized via SPS process. The effect of CaF_2_ on the structure of TSC-CF was investigated, and its friction and wear properties at 25–800 °C were explored.

## 2. Experiment

### 2.1. Preparation of Samples

All of the samples were prepared by SPS (Shanghai Chenhua Electric Furnace Co., Ltd., Shanghai, China). The composites were fabricated using powders of Ti_3_SiC_2_ (Jinhezhi Materials Ltd., Beijing, China) and CaF_2_ (Xinshengshi Chemical Technology Co., Ltd., Shanghai, China). The purity of the powder directly affected the performance of the prepared samples, so Ti_3_SiC_2_ powder with a purity of 98% and CaF_2_ powder with a purity of 98.5% were used for sample preparation. For the particle size, the sample sintered with fine-grained Ti_3_SiC_2_ powder had smaller grains and had better mechanical properties and wear resistance. Therefore, Ti_3_SiC_2_ powder with a particle size of 3 μm was selected. Additionally, CaF_2_ powder with overly small particles is not conducive to uniform powder mixing, so the CaF_2_ powder with a particle size of 74 μm was chosen. The volume fraction of CaF_2_ in Ti_3_SiC_2_-CaF_2_ composite powder was 15.

The Ti_3_SiC_2_-CaF_2_ composite powder was sintered at 1200, 1250, 1300 and 1350 °C, respectively. The heating rate and pressure were 50 °C/min and 35 MPa. The holding time was 5 min at maximum temperature, and then cooling to 1000 °C, followed by holding at 1000 °C for 15 min. For the purpose of comparison, pure Ti_3_SiC_2_ bulk materials (abbreviated as TSC) were sintered under the same conditions.

### 2.2. Mechanical Properties

The relative density of the composite material was determined by using Archimedes’ principle [26]. Micro-hardness was measured by a MH-5-VM Micro-hardness Tester (Shanghai Hengyi Technology Co., Ltd., Shanghai, China). A load of 4.9 N and a dwell time of 10 s were used. Flexural strength and compression strength were measured with a SANS-CMT5205 universal material tester (Shenzhen New Sansi Material Testing Co., Ltd., Shenzhen, China). The flexural strength and compression strength were measured with a universal testing machine. the samples sizes for flexural strength and compression strength were 3 mm high × 4 mm wide × 20 mm long and Φ 5 mm × 12.5 mm, respectively.

### 2.3. Friction and Wear Test

The friction and wear experiments used a pin-on-disk configuration (THT01-04015, CSM Instruments SA, Peseux, Switzerland). The pin was made of Ti_3_SiC_2_ and Ti_3_SiC_2_/ CaF_2_ sample with a size of Φ6 mm × 12 mm. The disk was made of Inconel 718 alloys with a size of Φ32 mm × 8 mm. The sliding velocity, normal load and sliding distance were 0.1 m/s, 5 N and 200 mm, respectively. The friction coefficient was automatically collected during the experiment. The volume loss method was used to calculate the wear volume. The cross-sectional area of the wear track on Inconel 718 disk was measured using optical microscopy and the dual-mode three-dimensional surface profiler (NanoMap-D, Columbus, OH, USA).

### 2.4. Analysis

The morphologies of worn surfaces were observed by a JSM-5600LV scanning electron microscopy (SEM, JEOL, Tokyo, Japan) which was equipped with energy dispersive spectroscopy (EDS). The X-ray photoelectron spectroscope (XPS, PHI-5702, Physical Electronics Corporation, Chanhassen, MN, USA) was used for the determined of elements chemical states on the worn surfaces.

## 3. Results

### 3.1. Phase Composition and Microstructure

**Figure 1:**
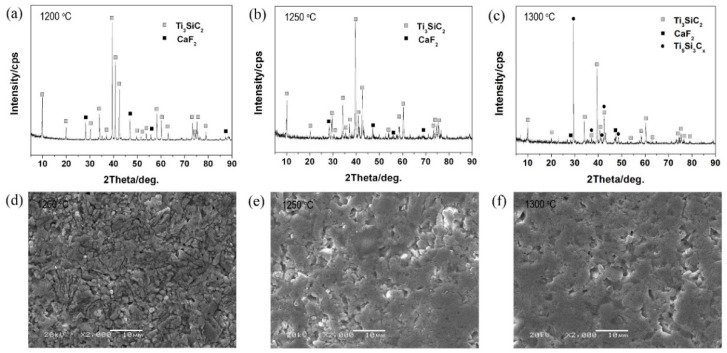
XRD pattern and SEM images of TSC-CF composites.

The XRD results (Figure 1) showed that the composites (85 vol.% Ti_3_SiC_2_-15 vol.%CaF_2_) sintered at 1200 and 1250 °C were mainly composed of Ti_3_SiC_2_ and CaF_2_, indicating that no reactions occurred between them and that Ti_3_SiC_2_ did not decompose. At 1300 °C, besides Ti_3_SiC_2_ and CaF_2_, diffraction peaks of Ti_5_Si_3_C_x_ were detected. This new phase was guessed to form due to the escapement of Si atoms from the crystal lattice of Ti_3_SiC_2_. The Si atoms moved along the base plane of the Ti_3_SiC_2_ crystal and then left the grain boundary at high temperatures [2]. After the escapement of Si atoms, the Ti_3_C_2_ framework was left, forming TiC_x_ because of the relaxation of Ti_3_C_2_ and the rearrangement of C atoms (Ti_3_SiC_2_→Si + Ti_3_C_2_(TiC_x_)). On the other hand, Si atoms reacted with Ti_3_C_2_ nearby to form Ti_5_Si_3_C_x_ (Ti_3_C_2_(TiC_x_) + Si→Ti_5_Si_3_C_x_). Finally, Si atoms combined with Ti_3_SiC_2_ to form Ti_5_Si_3_C_x_ (Ti_3_SiC_2_ + Si→Ti_5_Si_3_C_x_) [27]. Therefore, Ti_5_Si_3_C_x_ existed in the TSC-CF composite mainly due to the incomplete decomposition of Ti_3_SiC_2_. In comparison with our previous study, the addition of CaF_2_ promoted the decrease in decomposition temperature of Ti_3_SiC_2_ from 1350 to 1300 °C. SEM results indicated the grains of Ti_3_SiC_2_ in TSC-CF composites were granular. Additionally, compared with the original Ti_3_SiC_2_ powder, the grains of Ti_3_SiC_2_ in the TSC-CF composite were obviously larger, and their size increased with the sintering temperature.

### 3.2. Mechanical Properties

**Figure 2:**
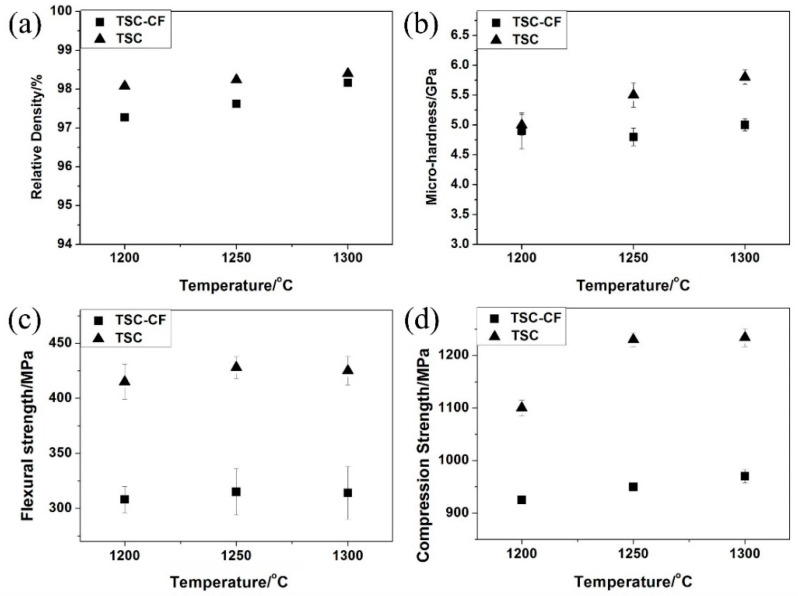
The (**a**) relative density, (**b**) micro-hardness, (**c**) flexural strength and (**d**) compression strength of TSC and TSC-CF at different temperatures.

As temperature increased, the relative density and bending strength of TSC did not change significantly, which were about 98.24% and 422.67 ± 6.8 MPa, respectively. As the sintering temperature of TSC increased, its hardness increased from 5 to 5.8 GPa, and its compressive strength rose from 1100 to 1234 MPa. As for the TSC-CF composite, both relative density and compressive strength increased with the sintering temperature. Additionally, the bending strength and hardness of TSC-CF composites were insensitive to the sintering temperature, and remained at 4.9 ± 0.1 GPa and 312.3 ± 3.79 MPa, respectively. In comparison, the relative density, hardness, compressive strength and flexural strength of the TSC-CF composite were lower than those of TSC, due to the addition of CaF_2_. It is shown in Figure 2 that both TSC and TSC-CF composites had better mechanical properties at 1250 °C.

**Figure 3:**
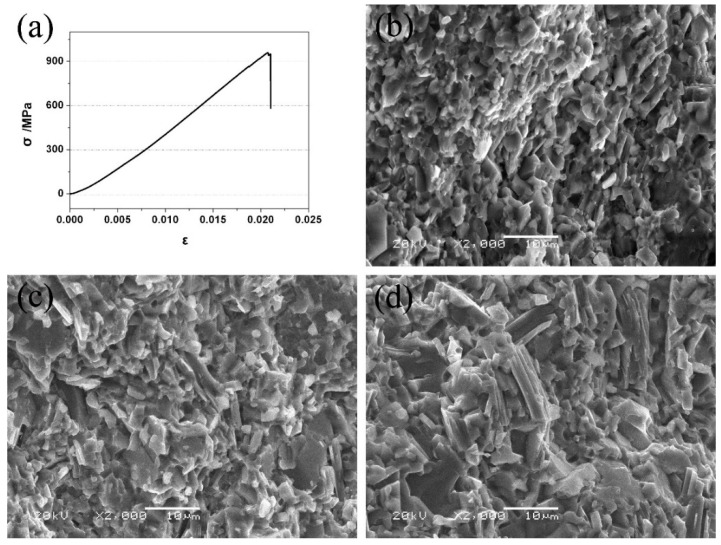
(**a**) Compressive stress–strain curve of the TSC-CF composite exposed to different sintering temperatures, and cross-sectional maps after the three-point bending test: (**b**) 1200 °C, (**c**) 1250 °C and (**d**) 1300 °C.

As seen in Figure 3a, TSC-CF composites only underwent elastic deformation before fracture, similarly to TSC. It was deduced that the addition of CaF_2_ did not change the fracture mode of Ti_3_SiC_2_. Additionally, the brittle fracture behavior at room temperature was also found in the bending fracture morphology of the TSC-CF composites (see Figure 3b–d), and a large number of Ti_3_SiC_2_ grains were distributed in a disordered manner, which was visible from a macroscopic view.

### 3.3. Friction Coefficients and Wear Rates

Both TSC and TSC-CF sintered at 1250 °C had better mechanical properties, so their friction and wear properties against Inconel 718 alloy at 25–800 °C were investigated for comparison purposes.

**Figure 4:**
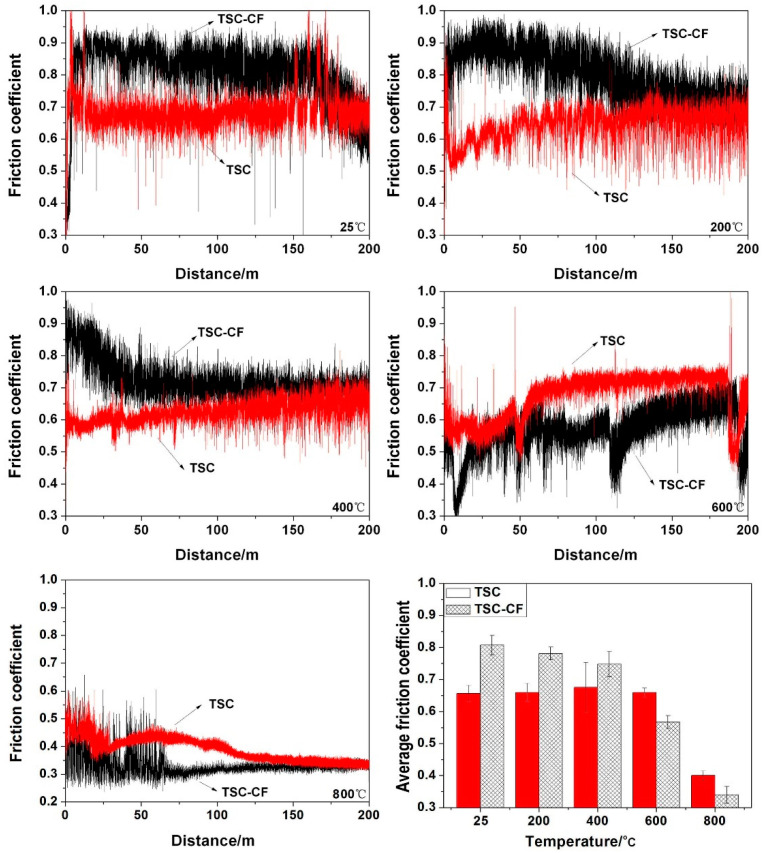
The friction coefficients of the TSC and TSC-CF with different temperature.

The relationship between the friction coefficients of TSC/Inconel 718 and TSC-CF/Inconel 718 pairs with sliding distance and their average friction coefficients at different friction test temperatures are shown in Figure 4. The friction coefficient of the TSC/Inconel 718 alloy pair had a large fluctuation at 25–200 °C and was relatively stable above 400 °C. However, the friction coefficient of the TSC-CF/Inconel 718 alloys pair greatly fluctuated at 25–600 °C and kept stable after 800 °C. TSC/Inconel 718 had no obvious running-in period during the friction process. TSC-CF/Inconel 718 had an obvious running-in period at 800°C. As the sliding temperature increased from 25 to 800 °C, the average friction coefficient of the TSC-CF/Inconel 718 alloys pair showed a downward trend (from 0.81 to 0.34). In comparison, the average friction coefficient of TSC-CF was higher than that of TSC at 25–400 °C, and it was lower than that of TSC at above 600 °C, owing to the addition of CaF_2_. In other words, CaF_2_ played had a certain self-lubricating effect above 600 °C.

**Figure 5:**
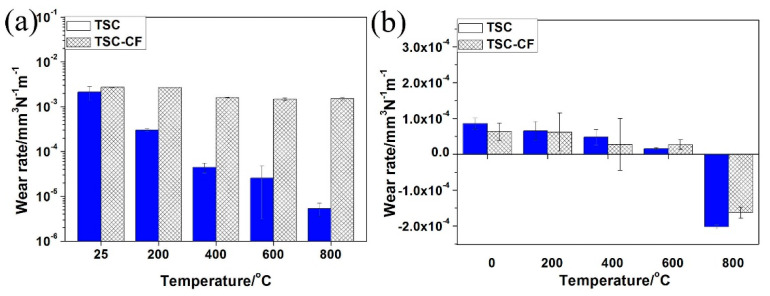
The wear rates of the (**a**) pin and (**b**) disk of the TSC/Inconel 718 and TSC-CF/Inconel 718 alloy tribo-pair.

As shown in Figure 5, the wear rate of the TSC pin in the TSC/Inconel 718 pair gradually decreased to 10^−6^ mm^3^/Nm from 25 to 800 °C. The wear rate of the TSC-CF pin in the TSC-CF/Inconel 718 pair was insensitive to the temperature (25–800 °C) and remained at 10^−3^ mm^3^/Nm. In comparison, the wear rate of the TSC-CF pin was greater than that of TSC at 25–800 °C. The wear resistance of the TSC-CF composite was lower than that of TSC, which might have resulted from the lower hardness of the TSC-CF composite (see Figure 4). As we all know, the wear resistance of a material largely depends on the hardness of the material. When sliding against TSC, the wear rate of the disc gradually decreased as the temperature increased from 25 to 600 °C, and remained at a magnitude of 10^−5^ mm^3^/Nm. However, negative wear occurred at 800 °C for the Inconel 718 alloy disc in the TSC/Inconel 718 pair. When grinding with TSC-CF, the wear rate of the Inconel 718 disc was similar to that of TSC.

In short, though the wear resistance of TSC-CF composites was worse than that of TSC at 25–800 °C, the TSC-CF composites exhibited better self-lubricating properties than TSC at 600–800 °C. Therefore, CaF_2_ was effective for using Ti_3_SiC_2_ as a solid lubricant at 600–800 °C.

### 3.4. Morphologies and Compositions of Worn Surfaces

**Figure 6:**
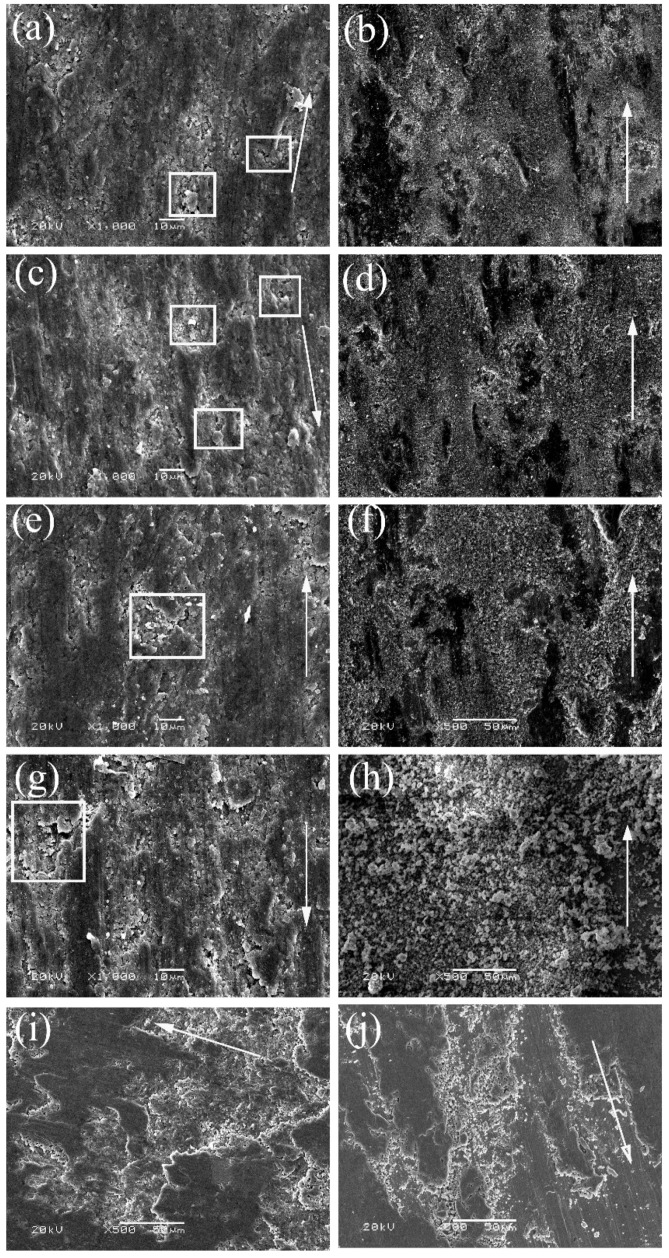
Worn surface of TSC-CF /Inconel 718 alloy tribo-pair: (**a**) TSC-CF at 25 °C, (**b**) Inconel 718 alloy at 25 °C, (**c**) TSC-CF at 200 °C, (**d**) Inconel 718 alloy at 200 °C, (**e**) TSC-CF at 400 °C, (**f**) Inconel 718 alloy at 400 °C, (**g**) TSC-CF at 600 °C, (**h**) Inconel 718 alloy at 600 °C, (**i**) TSC-CF at 800 °C, (**j**) Inconel 718 alloy at 800 °C.

The wear morphology of the pin and disc in the TSC-CF/Inconel 718 pair at 25–800 °C is shown in Figure 6. It shows that the wear surface of the TSC-CF composite was similar to that of Ti_3_SiC_2_. At 25–400 °C, many pits were seen on the wear surface of TSC-CF composite, which were caused by the pulling out and shedding of Ti_3_SiC_2_ grains (see Figure 6a–c) during sliding, making its wear surface relatively rough. The Ti and Si elements from the TSC-CF composite (see d, e, f in Table 1) were found on the Inconel 718 alloy’s surface (see Figure 6d–f) at 25–400 °C, indicating that one-way transfer of materials happened from the TSC-CF composite to the Inconel 718 alloy. The pulling out of the crystal grains and the shedding of the mechanical mixed layer contributed to the abrasive wear for TSC-CF/Inconel 718 pair at 25–400 °C.

As shown in Figure 6g, a plastic flow characteristic, similar to those of metal materials, was detected on the TSC-CF composite’s surface at 600 °C. However, the one-way transfer of materials from the TSC-CF composite to the Inconel 718 alloy still existed for the Inconel 718 alloy disc at 600 °C. Therefore, mechanical wear on the TSC-CF composite’s surface still occurred at 600 °C. At 800 °C, the plastic flow of the TSC-CF composite was more obvious, and its surface was very smooth. Additionally, there was the one-way transfer of material from TSC-CF to the Inconel 718 alloy.

**Figure 7:**
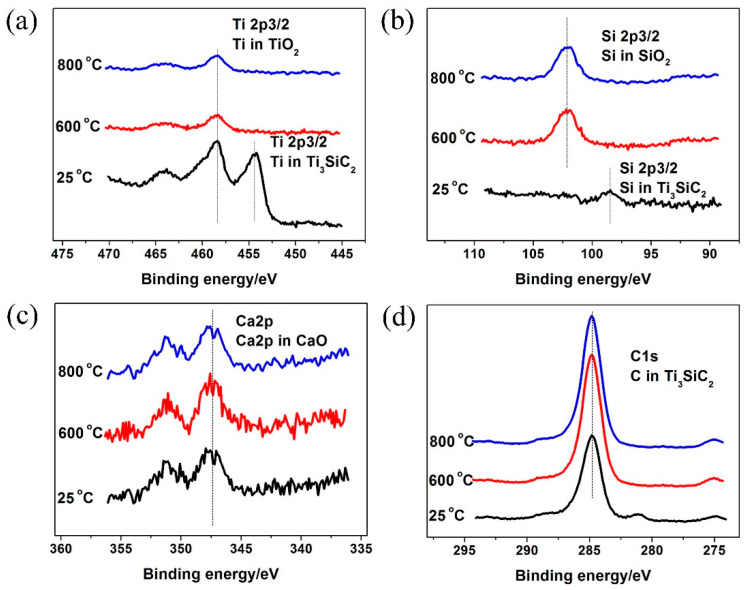
XPS for (**a**) Ti2p, (**b**) Si2p, (**c**) Ca2p and (**d**) C1s on the worn surface of TSC-CF at different temperatures.

The XPS analysis of Ti2p, Si2p, Ca2p and C1s on the TSC-CF’s wear scar surface in the TSC-CF/Inconel 718 alloy pairs at 25–800 °C is shown in Figure 7. At 25 °C, Ti existed in Ti_3_SiC_2_ and TiO_2_ on the worn surface of TSC-CF, and Si and Ca existed in Ti_3_SiC_2_ and CaO, respectively. At 600 °C and 800 °C, TiO_2_, SiO_2_ and CaO existed on the surface of the friction layer of TSC-CF. It was deduced that friction oxidation reactions occurred on the friction surface of TSC-CF during the sliding.

## 4. Discussion

### 4.1. The Temperature’s Effects on the Tribological Properties of Ti_3_SiC_2_/CaF_2_

The temperature affected the friction coefficient and wear rate of TSC-CF composites. As temperature increased (25–800 °C), the friction coefficient of TSC-CF decreased. Additionally, the tribo-chemical reaction (mainly the oxidation of Ti, Si and Ca) in the friction process was a key factor affecting the tribological performance of TSC-CF composites. At 25 °C, Ti existed in the forms of Ti_3_SiC_2_ and TiO_2_, due to incomplete oxidation. Simultaneously, Ca was oxidized to CaO, and Si did not oxidize at 25 °C, on the friction surface of TSC-CF. The oxide generation rate was less than its consumption rate, so the oxide layer could not be formed. At above 600 °C, the formation of an oxide layer benefited from the increase in temperature, preventing direct contact between the TSC-CF composite and Inconel 718 alloy and reducing the shedding of Ti_3_SiC_2_ grains and material transfer. Furthermore, the oxide layers formed at elevated temperatures exhibited more obvious plastic flow and less brittle fracturing, and were easier to shear, thereby contributing to the low friction of the TSC-CF composite at 600 and 800 °C.

### 4.2. Competition of Tribo-oxides

**Figure 8:**
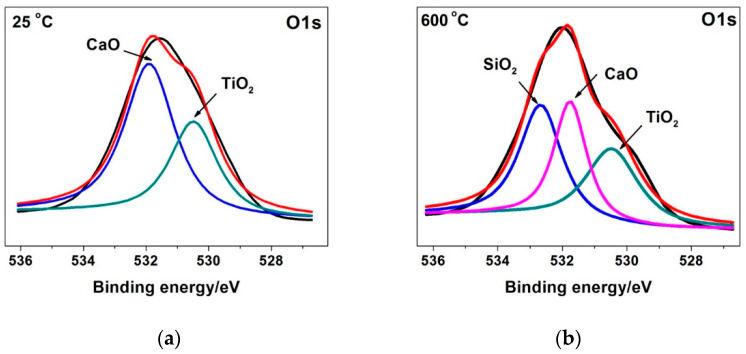
XPS spectra of O1s of Ti_3_SiC_2_/CaF_2_ pin surface at (**a**) 25 °C and (**b**) 600 °C.

Three kinds of oxides (namely, Ti, Si and Ca oxides) on the TSC-CF composite’s worn surface were detected. At 25 °C, only TiO_2_ and CaO were found on the worn surface of TSC-CF, and the content of CaO was relatively greater than that of TiO_2_. At 600 °C, an oxide film consisting of TiO_2_, SiO_2_ and CaO was examined on the wear surface of TSC-CF (see Figure 8). The transformation of these oxide species was due to the friction oxidation during the friction process, which in turn affected the friction and wear performance of the TSC-CF/Inconel 718 pair.

### 4.3. Formation of Oxide Layer

When two surfaces are sliding against with each other, the work due to friction is converted into heat, causing an increase in temperature at the sliding interface. Then, the elevated temperature changes the mechanical properties of the friction surfaces, causing oxidation or even melting, all of which can cause changes in the friction coefficient and wear rate. Huang Z Y et al. [28]. pointed out that the oxidation reaction occurring on the surface of Ti_3_SiC_2_ originates from frictional heat, and the generation and consumption (due to wear) of the oxides occurred on the friction surface. If the generation rate was greater than the consumption rate, an oxide layer was formed. However, when the generation rate was less than the consumption rate, an oxide layer was formed or the formed oxide layer was destroyed. In fact, the test temperature for the friction experiment was also a positive factor for affecting the formation of oxides. The higher the temperature, the more favorable the formation of oxides.

At 25 °C, incomplete tribo-chemical reactions occurred on the TSC-CF composite’s worn surface, only generating TiO_2_ and CaO. However, the frictional heat at 25 °C was not enough to provide enough energy to promote continuous oxidation. Therefore, the oxide generation rate was lower than the consumption rate, and could not result in an oxide layer on the TSC-CF composite’s surface due to the friction-reducing and anti-wear effects at 25 °C. In addition, the TSC-CF composite did not show plastic flow on its sliding surface, so the tribology properties of the TSC-CF composite at 25 °C were not improved. Compared with Ti_3_SiC_2_, the oxide particles generated on the TSC-CF composite’s worn surface could not form an oxide layer, and they easily fell off from the wear surface during sliding, causing more severe abrasive wear than Ti_3_SiC_2_. Thus, the friction and wear of TSC-CF composite were higher than those of Ti_3_SiC_2_. Above 600 °C, the TSC-CF composite’s wear surface experienced tribo-chemical reactions, and three kinds of oxides (including TiO_2_, SiO_2_ and CaO) were generated. Then, the frictional heat could provide enough energy to promote the continuous generation of oxides, forming an oxide layer of TSC-CF. Moreover, the formed oxide layer exhibited obvious plastic flow, making it easier to shear. Additionally, this oxide film prevented direct contact between TSC-CF and Inconel 718 alloy, and reduced shedding of Ti_3_SiC_2_ grains and material transfer. Therefore, TSC-CF showed little friction behavior.

An interesting phenomenon in this study was the oxidation of Si in the friction layer. At 25 °C, the formation rate of SiO_2_ was lower than its consumption rate. The detection of silicon oxide on the wear surface of TSC-CF was irrefutable evidence of the occurrence of tribochemical reactions. However, the TSC-CF wear surface did not form an oxide layer at 25 °C, so it exhibited a high friction coefficient. At 800 °C, complete tribochemical reactions occurred and a large area of plastic flow appeared on the friction surface of TSC-CF. The elevated temperature facilitated the maintenance of the oxide layer on the friction surface. This oxide layer prevented the direct contact between TSC-CF and Inconel 718 alloy, reducing the shedding of Ti_3_SiC_2_ grains and material transfer. The oxide layer on the TSC-CF friction surface showed significant plastic flow and it was easier to shear. Furthermore, its brittle fracture was obviously reduced. All these factors made TSC-CF reduce friction and have high wear resistance.

## 5. Conclusions

The tribological behavior of the Ti_3_SiC_2_/CaF_2_-Inconel 718 tribo-pair strongly depends on the environmental temperature. Differences in temperature cause changes in friction oxides. Tribo-oxidation reactions mainly included the oxidation of Ti, Si and Ca. At 25 °C, only TiO_2_ and CaO were formed. At 600 °C and above, the tribo-oxidation products included TiO_2_, SiO_2_ and CaO. Therefore, Ti_3_SiC_2_/CaF_2_ exhibited better self-lubricating properties than Ti_3_SiC_2_ at 600 and 800 °C. The addition of CaF_2_ was effective at improving the self-lubricating performance of Ti_3_SiC_2_ at 600 and 800 °C.

## Figures and Tables

**Table 1:** Atomic percentages in Figure 6.

Position	Sample	Temperature/°C	Atomic Percentages
a	TSC-CF pin	25	30.7%Ti, 13.2%Si, 41.3%C, 3.4%Al, 4.1%Ca, 7.3%F
c	TSC-CF pin	200	27.0%Ti, 11.2%Si, 44.1%C, 3.3%Al, 4.6%Ca, 9.8%F
e	TSC-CF pin	400	27.4%Ti, 10.1%Si, 47.0%C, 3.0%Al, 4.3%Ca, 8.2%F
g	TSC-CF pin	600	32.3%Ti, 9.5%Si, 42.9%C, 2.5%Al, 3.4%Ca, 9.4%F
i	TSC-CF pin	800	34.3%Ti, 8.9%Si, 41.3%C, 3.3%Al, 4.1%Ca, 8.1%F
b	Inconel 718 disk	25	15.6%Ti, 7.0%Si, 33.8%C, 1.6%Al, 2.0%Ca, 20.2%O, 9.3%F, 5.3%Ni, 2.7%Cr, 2.5%Fe
d	Inconel 718 disk	200	16.7%Ti, 7.1%Si, 32.8%C, 1.8%Al, 2.0%Ca, 23.1%O, 8.8%F, 3.8%Ni, 2.1%Cr, 1.8%Fe
f	Inconel 718 disk	400	13.7%Ti, 6.1%Si, 30.6%C, 2.0%Al, 1.6%Ca, 26.8%O, 7.6%F, 6.1%Ni, 3.0%Cr, 2.5%Fe
h	Inconel 718 disk	600	14.3%Ti, 5.2%Si, 12.7%C, 1.7%Al, 1.6%Ca, 59.8%O, 3.4%F, 0.7%Ni, 0.6%Fe
j	Inconel 718 disk	800	15.3%Ti, 5.8%Si, 11.2%C, 1.7%Al, 1.6%Ca, 59.6%O, 3.3%F, 0.9%Ni, 0.6%Fe

## Data Availability

The data presented in this study are available within the article.
